# Phylogenetic position and age of Lake Baikal candonids (Crustacea, Ostracoda) inferred from multigene sequence analyzes and molecular dating

**DOI:** 10.1002/ece3.3159

**Published:** 2017-08-01

**Authors:** Ivana Karanovic, Tatiana Ya. Sitnikova

**Affiliations:** ^1^ Department of Life Science College of Natural Science Hanyang University Seoul South Korea; ^2^ Institute for Marine and Antarctic Studies University of Tasmania Hobart Tas Australia; ^3^ Limnological Institute Siberian Branch Russian Academy of Sciences Irkutsk Russia

**Keywords:** ancient lakes, evolutionary history, fossil calibration, molecular clock

## Abstract

With 104 endemic species family Candonidae is one of the most diverse crustacean groups in Lake Baikal, yet their phylogenetic relationships and position in the family have not been addressed so far. Here, we study the phylogenetic position of Baikal candonids within the family and their evolutionary history using molecular markers for the first time since their original description. We choose 10 Baikal and 28 species from around the world, and three ribosomal RNA‐s (18S, 28S, and 16S), and analyze individual and concatenated datasets using Bayesian Inference in MrBayes and BEAST. For molecular divergence time estimates, four fossil records are used to calibrate the root and three internal nodes. The 28S dataset is tested under the strict molecular clock, while for other data we use relaxed clocks. Resulting trees show incongruence between molecular and fossil divergence time estimates, with the former suggesting older ages. Strict molecular clock analysis results in narrower node age confidence intervals and younger time estimates than other analysis. All trees support at least two candonid lineages in Baikal, with two independent colonization events, and 28S suggests a major radiation between 12 and 5 Mya. This divergence time estimate mostly agrees with another, unrelated, ostracod group in the lake and other lake animals as well. Baikal candonid clades show a close phylogenetic relationship with Palearctic lineages, but their deep divergence is indicative of separate genera. Results also suggest a monophyly of tribes that today live exclusively in subterranean waters, and we offer several hypotheses of their evolutionary history.

## 1 INTRODUCTION

From dinoflagellates (Annenkova, 2013) to seals (Palo & Väinölä, 2006), Lake Baikal is a place of exceptional biodiversity. Geological history of Baikal covers the period of 25–30 million years (Müller et al., 2001; Sherbakov, 1999), and for the greatest part it was represented by shallow basins slowly unifying together, first southern and central, and finally joined by the north basin (0.8–0.5 million years ago). The single ultradeep reservoir (over 1000 meters) formed relatively recently, 500,000–150,000 years ago (Logachev, 2003; Mats, 2001; Popova et al., 1989). Climate shifts from subtropical to continental (Popova et al., 1989), Pleistocene glaciations, as well as evolution of abyssal depths promoted rapid speciation in Baikal (Khursevich et al., 2001). Over 2,500 species have been recoded so far (Timoshkin, 2001), and more than half of the known species are endemic to this lake; many lineages are represented by series of “flocks of closely related species” (Martens, Coulter, & Goddeeris, 1994; Timoshkin, 2001).

In comparison with other ancient lakes and surface freshwater ecosystems in general, Lake Baikal has the highest proportion of crustaceans in its fauna (32%; see Martens & Schön, 1999). Amphipods are the most diverse crustacean group, with nearly 300 species (Takheev, 2000). The second largest are ostracods; according to Martens (1994) there are 174 species and subspecies here, of which 90% are endemic. Lake Tanganyika, in comparison, has 64 ostracod species and subspecies, but a slightly higher endemicity (94%) (Martens, 1994), and far more endemic genera than Baikal. However, the latter is a matter of the current systematics and previous taxonomic decisions, and does not necessarily reflect phylogeny of the group (Martens et al., 1994). Both Baikal and Tanganyika have some nonendemic taxa. In Lake Baikal, Palearctic taxa live in the top 2 m of the littoral only and do not penetrate beyond this zone; only very few Baikal endemics live sympatrically with these Palearctic species (Mazepova, 1994). Present data indicate that in Lake Tanganyika many endemics abound in the upper‐littoral of the lake (at depths of 0.5 m and less) (Martens, 1994).

Lake Baikal is populated by two ostracod suborders: Cytherocopina and Cypridocopina. The former, predominantly a marine group, is represented by two genera: *Limnocythere* Brady, 1867 (one species) and *Cytherissa* Sars, 1925 (47 species and 10 subspecies). The latter is represented by the family Candonidae, exclusively a freshwater group, classified into three genera: *Candona* Baird, 1845 (48 species and five subspecies); *Pseudocandona* Kaufmann, 1900 (27 species and three subspecies); and *Baicalocandona* Mazepova, 1976 (11 species and 10 subspecies). Only *Baicalocandona* is endemic to Lake Baikal, while the other four genera have primarily Holarctic distributions. The family Candonidae today numbers about 500 Recent species (Karanovic, 2012; Martens & Savatenalinton, 2011), of which almost a half live either in Lake Baikal or in the subterranean waters of Western Australia (Karanovic, 2007).

A majority of Baikal candonids (and also *Cytherissa*) were described in two main publications: Bronstein (1947) and Mazepova (1990). These descriptions, although missing some important taxonomic information, revealed a great morphological diversity and indicated that Baikal candonids need to be revised and probably subdivided into several genera (Danielopol, Baltanás, Morocutti, & Österreicher, 2011; Karanovic, 2007, 2012). Karanovic (2007) provided a phylogenetic reconstruction of the family Candonidae based on morphological characters alone, erecting several new tribes, of which the largest one (the nominotypical tribe *Candonini*) remained paraphyletic. This is partly due to a high morphological diversity of Baikal candonids belonging to this tribe.

Thanks to their well calcified shell, ostracods are one of the most abundant microfossil groups. More than 80% of species are known only from the fossil record, which stretches back to Ordovician (Siveter, Briggs, Siveter, & Sutton, 2010). One of the most reliable characters for discrimination of higher systematic ranks, such as families, in the fossil record is the adductor muscle scar imprint on the shell. Shell ornamentation and shape are used for lower taxonomic units. The candonid shell is generally poorly ornamented and with high intrageneric shape variability, which may pose a problem in fossils identification. The record of Candonidae from the Upper Carboniferous is dubious because of very poorly preserved shells, with undistinguishable pattern of muscle scar imprints (Sohn, 1975, 1977). According to Danielopol et al. (2011), the oldest Candonidae ostracod dates back to Early Jurassic and is attributed to *Septacandona* Cabral & Colin, 2002 from Portugal (Cabral & Colin, 2002). The exact number of fossil Candonidae is hard to corroborate partly due to a great variability in the carapace shape, but also because of discrepancy between paleontological and neontological systematics of the family. For example, Krstić (2006) provided an overview of the Pliocene ostracods from the Pannonian plane and divided the family into 11 tribes, separating genera which are very closely related based on neontological data.

There are numerous caveats for the use of fossil record for molecular clock calibrations (Parham & Irmis, 2008), but this method has nevertheless been widely applied to aid divergence time estimations in various groups (see Gandolfo, Nixon, & Crepet, 2007; Warnock, Parham, Joyce, Lyson, & Donoghue, 2014). Despite an abundant ostracod fossil record, their age is rarely used in molecular clock calibrations. In addition, a study based on 18S rRNA stipulated a high incongruence between fossil and molecular divergence time estimates in this group, partly due to the controversial taxonomy of fossil ostracods (Tinn & Oakley, 2008). Consequently, studies attempting to estimate divergence times in ostracods mostly applied universal invertebrate COI molecular clock rates proposed by Wilke, Schultheiß, and Albrecht (2009) (see Schön, Shearn, Martens, Koenders, & Halse, 2015), or rates calculated for some ostracod lineages (Schön, Martens, van Doninck, & Butlin, 2003) based on COI and ITS markers. The study of the evolutionary history and phylogenetic relationships of Lake Baikal and Lake Tanganyika Cytherocopina by Schön and Martens (2012) exerted several dating methods in order to compare the divergence times of this ostracod group in two ancient lakes. Using geological dates of the two lakes origins, fossil record, and Wilke's universal COI molecular clock, the authors settled with the last method which placed the origin of *Cytherissa* species flocks in Lake Baikal between 8 and 5.3 Mya, rather similar with the age estimates based on fossil record.

The age of *Cytherissa* in Lake Baikal is in accordance with other animal groups and shows that Baikal's diverse endemic fauna is young, but it may stem from ancient lineages (Hidding, Michel, Natyaganova, & Sherbakov, 2003). Overall, the highest species flock explosions happened in the post‐Pliocene ages, when the current ecological conditions established and abyssal parts of the lake expanded and became well oxygenated (Stelbrink et al., 2015). Animal groups mostly differ in the number of lake colonization events and in the evolutionary age of colonizers. In amphipods, molecular data suggested several independent colonization events of the lake, and subsequent diversifications (Macdonald, Yampolsky, & Duffy, 2005). In addition, invading lineages were much older than the lake itself and not even closely related (see Daneliya, Kamaltynov, & Väinölä, 2011; Sherbakov, 1999). Diversification of the Baikal endemic sculpin fishes started around 2‐3 Mya (Kontula, Kirilchik, & Väinölä, 2003), very similar to that of the limpet lineages (Stelbrink et al., 2015) and prosobranchian mollusk endemic family Baicalidae (Zubakov, Sherbakov, & Sitnikova, 1997). On the other hand, the pulmonate mollusks have a similar evolutionary scenario to the amphipod lineages (Starobogatov & Sitnikova, 1992). The age of Lake Baikal copepods was estimated to 20–25 Mya (Mayor, Sheveleva, Sukhanova, Timoshkin, & Kirilchik, 2010).

The aim of this research was to study evolutionary history and phylogeny of Baikal candonid ostracods, which has not been done so far. To address this problem, we use three molecular markers (18S rRNA, 28S rRNA, and 16S rRNA) and 38 Candonidae species, of which 10 are from Lake Baikal and include representatives of all three genera. We also want to verify whether the evolutionary history of Baikal candonids is congruent with *Cytherissa* and other animal groups in the lake. By conducting molecular divergence time analyzes on the concatenated dataset and on 18S rRNA and 28S rRNA separately, we will test weather different datasets with the same calibration points and ages render similar time estimates. In addition, our results will test if the divergence time estimates based on slowly evolving nuclear markers (such as 18S and 28S) are comparable to those based on *COI* (Schön & Martens, 2012).

## 2 MATERIAL AND METHODS

### 2.1 Collecting

Samples were taken from 11–15 m depths by SCUBA diving from the shore of Lake Baikal at Listvyanka (51°51′51.3″N 104°50′37.8″E) on September 12, 2015. Three bottom types were sampled: rock, mud, and sand. Ostracods were sorted alive on the spot and immediately fixed in 97% ethyl alcohol. Dissection and identification were performed with the aid of Zeiss Axiostar‐plus light microscope and Leica DM 2500 compound microscope, equipped with N‐Plan objectives. Scanning Electron Microscope (SEM) photographs were taken with a Hitachi S‐4700 at Eulji University (Seoul).

### 2.2 Nomenclature choices

In this study, we followed a recent revision of Cypridocopina (Hiruta, Kobayashi, Katoh, & Kajihara, 2016) based on the molecular phylogenetic analysis in which three Candonidae subfamilies, Candoninae, Paracypridinae, and Cyclocypridinae were all erected to the family level. In our analysis for each species we retained genera names in which they were originally described, unless a new combination has been proposed later on. For example, the genus *Typhlocypris* Vejdovský, 1882 is considered a senior synonym of *Pseudocandona* (see Karanovic, 2005), but not all species described in *Pseudocandona* have been given a new combination, so we abstained from doing this in the present publication. Namiotko, Danielopol, Meisch, Gross, and Mori (2014) redefined *Typhlocypris* to include a number of species originally described in *Pseudocandona*, none of which is part of our analysis. The same authors retained *Pseudocandona* for the rest of the species.

### 2.3 DNA extraction and amplification

In the first step of the DNA extraction, specimens were kept for 2–3 hr in distilled water. LaboPass Tissue Mini extraction kit (Cosmo Genetech Co., Ltd, Korea) was used in all further steps of extraction, following the manufacturer's protocol. Fragments of 28S were amplified using the primer pairs dd/ff, ee/mm, vv/xx from Hillis and Dixon (1991), of the 18S with primers from Yamaguchi (2003), and fragments of 16S were amplified with primers from Palumbi et al. (1996), all using a TaKaRa PCR Thermal Cycler Dice. For all amplifications, PCR reactions were carried out in 25 μl volumes, containing: 5 μl of DNA template, 2.5 μl of 10× ExTaq Buffer, 0.25 μl of TaKaRa Ex Taq (5 units/μl), 2 μl of dNDTP Mixture (2.5 mmol/L each), 1* *μl each primer, and 13.25 μl distilled H_2_O. The PCR protocol for 28S consisted of initial denaturation for 5 min at 94°C, 40 cycles of denaturation for 35 s at 95°C, annealing for 1 min at 50°C, extension for 1 min at 72°C. Final extension was at 72°C for 5 min. PCR settings for the amplification of 18S followed Yamaguchi (2003) for each corresponding primer pair. Settings for 16S consisted of initial denaturation at 94°C for 5 min, 35 cycles of denaturation for 30 s at 94°C, annealing for 30 s at 48°C, extension for 1 min at 72°C. Final extension was at 72°C for 10 min. The PCR products were electrophoresed on 1% agarose gels; if DNA was present the products were purified for sequencing reactions using the LaboPass PCR Purification Kit, following the guidelines provided with the kit. DNA was sequenced on an ABI automatic capillary sequencer (Macrogen, Seoul, South Korea) using the same set of primers always in both directions.

### 2.4 Molecular data analysis

All sequences were visualized using Finch TV version 1.4.0 (http://www.geospiza.com/Products/finchtv.shtml). BLAST (Altschul, Gish, Miller, Myers, & Lipman, 1990) analysis of GenBank database was used to check that the obtained sequences were ostracod in origin and not contaminants. Each sequence was checked for the quality of signal and sites with possible low resolution, and corrected by comparing forward and reverse strands. Sequences were aligned in MEGA 7 (Kumar, Stecher, & Tamura, 2016) with ClustalW (Thompson, Higgins, & Gibson, 1994) with extension penalty changed from default settings (6) to 1 for 28S dataset in order to allow alignment of homologous regions that were separated by expansion segments present in some taxa but not others. All alignments were manually checked and corrected where necessary. The 28S alignments were also checked with Gblock (Castresana, 2000), and ambiguous blocks were removed. We analyzed alignment of each gene and all three regions of 28S amplified with different primes (dd/ff, ee/mm, vv/xx) separately. In addition, we performed two analyzes of the concatenated dataset: one including all three genes, and the other with only three 28S fragments; the latter was used only in the divergence time estimations. In the concatenated datasets, some species datasets were composed of sequences acquired from different specimens in order to avoid missing data, and for our outgroup we combined 16S from a different, but closely related, species. Missing data in concatenated datasets were coded “?”. Recent simulations and empirical analyzes suggested that missing data in Bayesian phylogenetics are not themselves problematic, and that incomplete taxa can be accurately placed as long as the overall numbers of characters are large (Wiens, 2003; Wiens & Moen, 2008). Sequence differences within and between groups in each individual alignment, as well as in concatenated datasets, were calculated in MEGA 7 using simple p‐distance method. Sequences are divided into groups, defined by the genus they belong to. For the best fit evolutionary model program, jModelTest 2.1.6 (Darriba, Taboada, Doallo, & Posada, 2012; Guindon & Gascuel, 2003) was used with the Akaike information criterion (Hurvich & Tsai, 1989). Bayesian inference reconstruction in MrBayes v3.2.6 (Huelsenbeck & Ronquist, 2001; Ronquist & Huelsenbeck, 2003; Ronquist et al., 2012) was performed with the best fit model and priors for the base and state frequencies calculated by jModelTest. For the concatenated set, data were partitioned into five blocks corresponding to gene regions, each with its fixed priors. All analyzes ran with four chains simultaneously for two million generations in two independent runs, sampling trees every 200 generations. Of the four chains three were heated, and one was cold, the temperature value (“Temp” command in MrBayes) was 0.1 (default option). The results were summarized, and trees from each MrBayes run were combined with the default 25% burn‐in. A >50% posterior probability consensus tree was constructed from the remaining trees. For the choice of the outgroup we relied on the phylogeny published in Hiruta et al. (2016). As the relationships within Cypridoidea were not clearly resolved and Candonidae appears as a sister taxon to all other Cypridoidea, we decided on a representative of Cyclocyprididae, which used to be in the same family with Candoninae. For details of the number of original sequences, their sampling localities as well as for those downloaded from GenBank (Supplement 1).

Saturation test and likelihood ratio test for deviation from molecular clock of each separate dataset were performed with DUMBE5 (Xia, 2013), while for the concatenated datasets marginal model likelihood using stepping stone algorithm was applied to test molecular clock in MrBayes. After examining the consensus tree resulted from separate and concatenated analysis we chose four nodes to calibrate the molecular clock in the divergence time analysis performed in BEAST v1.8.3 (Drummond, Suchard, Xie, & Rambaut, 2012). Three analyzes were run as follows: concatenated dataset with all three genes, 18S dataset, and combined 28S dataset. The last differed from the first two in using strict clock model, while in the case of the first two we used uncorrelated relaxed (lognormal) clock. Otherwise in all three analyzes, GTR + G + I model (Rodríguez, Oliver, Marín, & Medina, 1990) was used for the site model and Calibrated Yule model (Heled & Drummond, 2011) for the tree priors. Priors for the node ages were all set with normal distribution. The root was calibrated based on the oldest Candonidae fossil with a mean of 180 Mya and standard deviation of 6 Mya, covering the period of the Early Jurassic. The three internal nodes were calibrated as follows: *Candona* origin with a mean of 80 Mya and standard deviation of 3.2 Mya, corresponding to the time of the first *Candona ssl*. fossils from the Upper Cretaceous (see Danielopol et al., 2011); *Pseudocandona* origin with a mean of 24 Mya and standard deviation of 2.6 Mya, corresponding to the time of the first *Pseudocandona ssl*. fossil from Late Oligocene/Early Miocene (Triebel, 1963); and *Trapezicandona* Schornikov, 1969 with a mean of 6 Mya and standard deviation of 1 Mya, corresponding to the time of the first *Trapezicandona* fossils from Late Miocene/Early Pliocene period (see Danielopol, 1968). All other priors were set to default program options. We conducted two independent runs for each analysis, each for 10,000,000 generations, sampling every 1,000 generations. Software Tracer (Rambaut, Suchard, Xie, & Drummond, 2014) was used for visualizing results of the BEAST analyzes and FigTree v1.4.3 for tree visualizations. We did not analyze 16S separately for the divergence time estimate, because of a very limited dataset.

**Figure 1:**
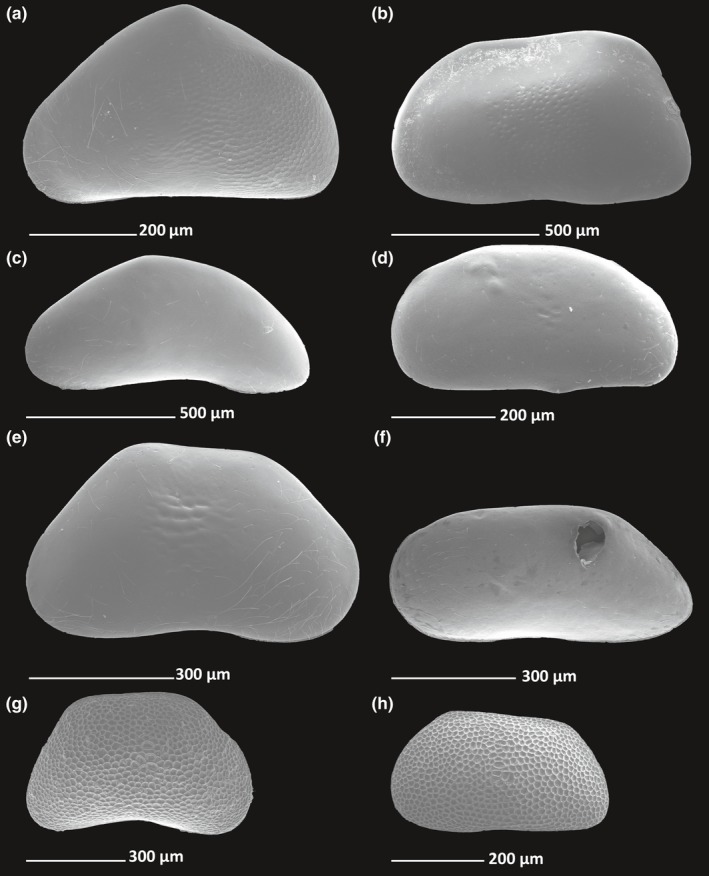
SEM images shells of Lake Baikal candonid representatives: (a), *Baicalocandona navitarum*; (b), *Candona directa*; (c), *Candona godlewski*; (d), *Candona orbiculata*; (e), *Candona rupestirs*; (f), *Candona spicata*; (g), *Pseudocandona sp. 1*;(h), *Pseduocandona* sp. 6

## 3 RESULTS

### 3.1 Taxonomy

The samples collected from the lake contained representatives of both Baikal ostracod groups: *Cytherissa* and various representatives of Candonidae. Of all candonid morphotypes found we were able to confidently identify the following species: *Baicalocandona navitarum* Mazepova, 1976; *Candona directa* Bronstein, 1947; *Candona godlewski* Mazepova 1984; *Candona orbiculata* Mazepova, 1990; *Candona rupestris* Mazepova, 1990; and *Candona spicata* Mazepova, 1982 (Figure 1a–f). Beside these six species, another four have been included in the analysis, but not identified to the species level as they were all at some of the juvenile stages. Two species were placed in *Pseudocandona* because they had strongly ornamented rectangular shells, typical for the Baikal Lake representatives of this genus (Figure 1g, h). *Candoninae 7* and *Candoninae 10* were left without any generic assortment. They both had a smooth carapace.

**Figure 2:**
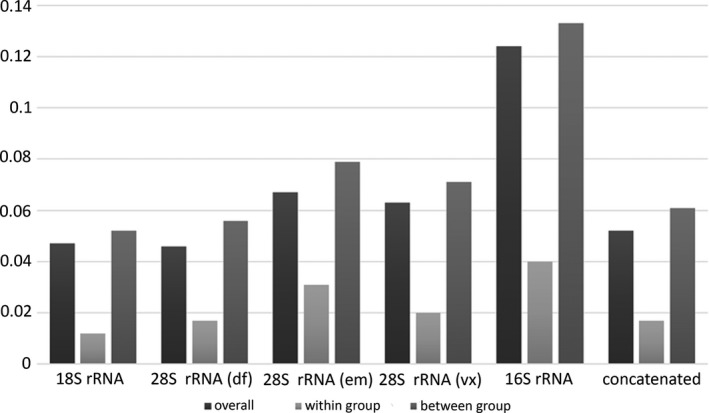
Pairwise *p*‐distances for individual and concatenated datasets

### 3.2 Sequence diversity

The concatenated dataset was 3302 base pairs long, and it included 50 taxa. Of the individual alignments, 18S dataset was the longest (1042 positions) and also included 50 terminals. The alignment of 16S was the shortest (554 base pairs), and had only 21 species. After the exclusion of ambiguous blocks, 28S alignments varied from 660 base pairs (em fragment) to 455 base pairs (df fragment). The vx primer pair was the most successful in amplifying the region, while df fragment was very difficult to amplify and only 34 sequences were analyzed. The amplification by em primer pair was relatively successful, but this was the most difficult dataset to aligned due to the long expansion segments present in several species. Although initially this alignment was very long (1,521 base pairs), after the Gblock analysis (Castresana, 2000) it was truncated substantially.

GTR (Rodríguez et al., 1990) (or its variations) with unequal rates among sites, with gamma distribution and invariable site (GTR + G + I) for 18S, 16S, 28S (df and vx fragments), but without invariable sites for 28S, em fragment was chosen as the best fit evolutionary model. Supplement 2 summarizes general information for each alignment and also includes the base and rate frequencies, proportion of invariable sites and gamma shape.

The results of pairwise distance analysis are shown in Figure 2. Within group means did not exceed 4% in any of the datasets. Between group means varied from 5% for 18S to 13% for 16S. Of the three fragments of 28S, em was the most variable, followed by vx, and df fragments. The results of the p‐distance analysis show that 16S is by far the fastest evolving gene, followed by 28S, and 18S, although there is little difference in values between the latter gene and the 28S df fragment.

**Figure 3:**
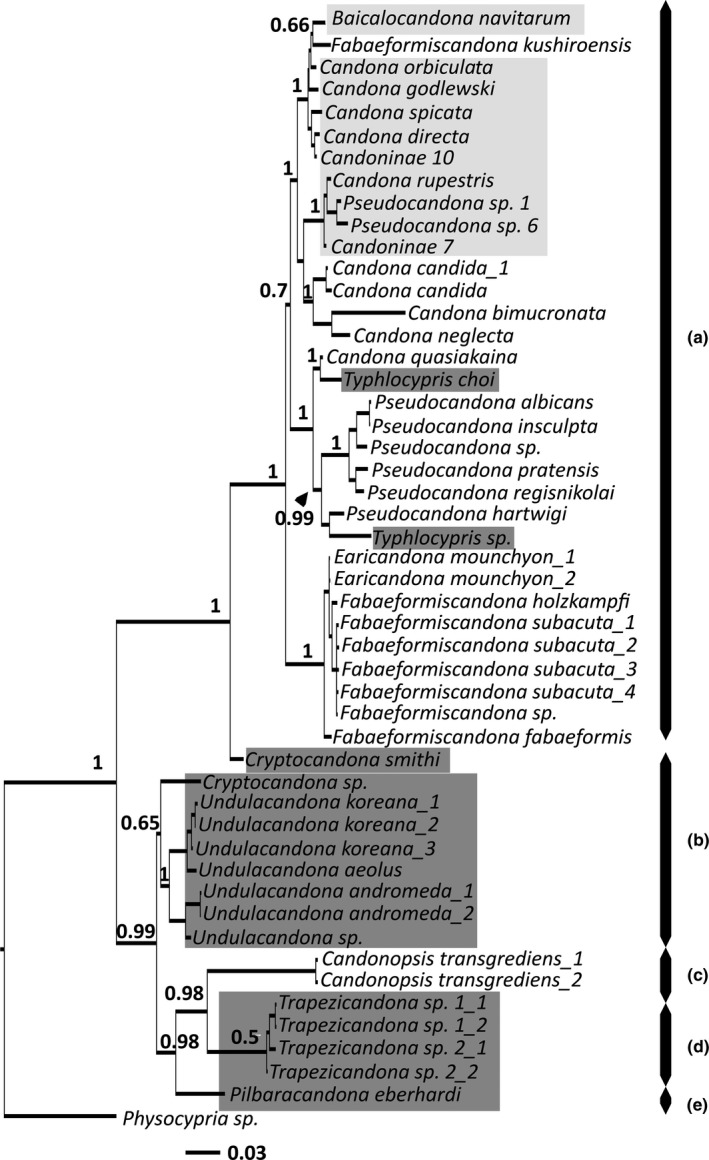
50% Majority role consensus molecular phylogenetic tree of the family Candonidae and an outgroup constructed from the concatenated dataset. Numbers on branches represent Bayesian posterior probability. Light gray shaded taxa are Lake Baikal candonids, dark gray shaded taxa are subterranean species, no shaded taxa are surface water species. Letters next to taxa denote individual tribes: (a) Candonini; (b) Cryptocandonini; (c) Candonopsini; (d) Trapezicandonini; (e) Humphreyscandonini

### 3.3 Phylogeny

After two million generation runs in MrBayes, the final standard deviation of split frequencies fell below 0.01 (for all datasets it was around 0.003) and the potential scale reduction factor was ~1.0 for all parameters, suggesting that convergence had been reached. All resulting consensus trees were rooted with the outgroup–*Physocypria biwaense* and *P. cf. biwaense*, or *P*. sp. in the case of 16S dataset analysis. Figure 3 illustrates the 50% consensus tree resulting from the analysis of the concatenated dataset. On this tree, Candonidae is strongly supported as a monophyletic group. The Candonidae clade can be broadly divided into two subclades, both with high posterior probability values: one containing 15 sequences equating to nine species, and the other which incorporates 34 sequences belonging to 28 species. The former clade contained four Candonidae tribes, proposed by Karanovic (2007): Cryptocandonini (letter “b”), Candonopsini (letter “c”), Trapezicandonini (letter “d”), and Humphreyscandonini (letter “e”). Candonopsini was a sister taxon to Trapezicandonini, while Humphreyscandonini was the sister taxon to these two. These relationships received a relatively high posterior probability support, while the clade consisting of the two Cryptocandonini genera (*Cryptocandona* Kaufamann, 1900 and *Undulacandona* Smith, 2011) did not have high posterior probability.

**Figure 4:**
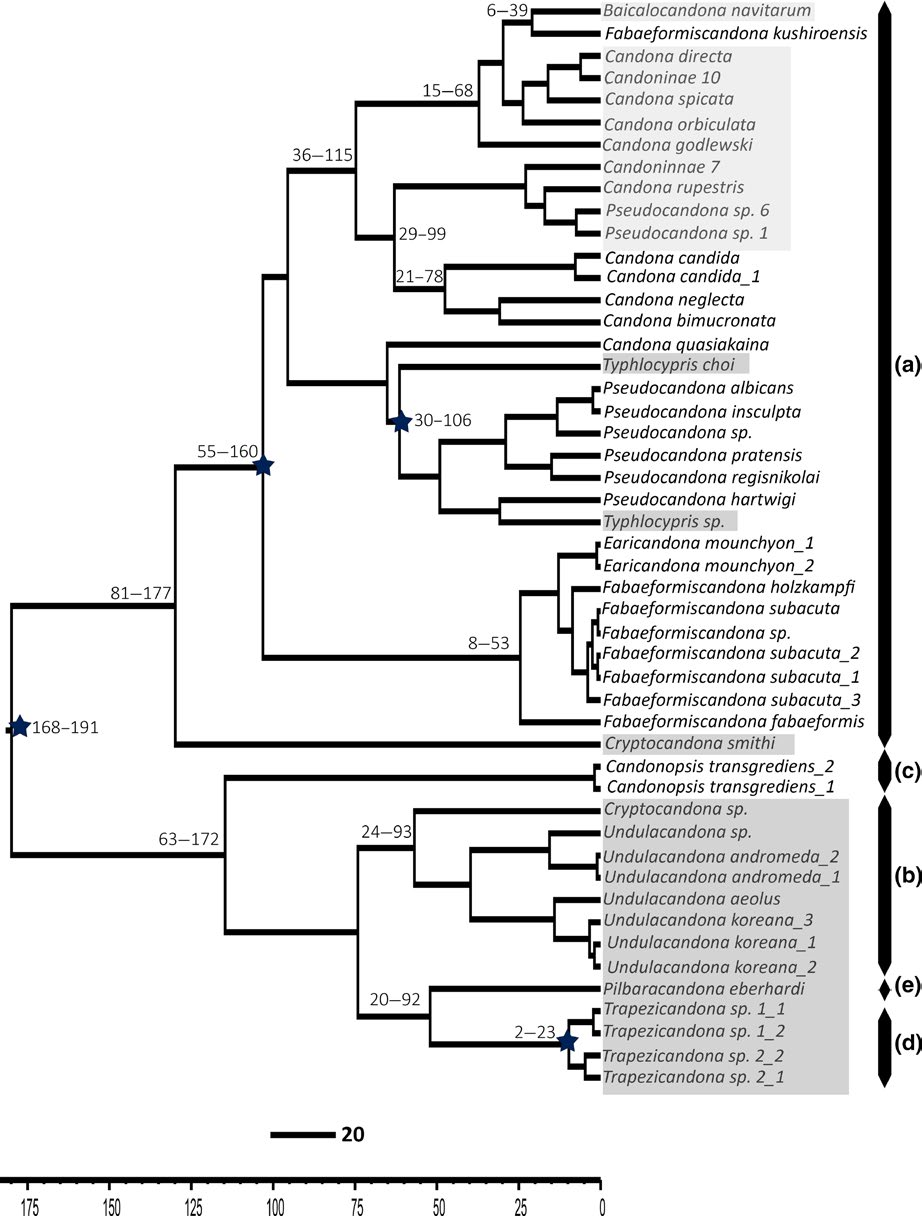
Molecular time divergence estimate tree of the family Candonidae constructed from the concatenated dataset. Stars represent nodes calibrated with fossil record. Numbers above branches represent 95% HPD intervals for particular node heights. Light gray shaded taxa are Lake Baikal candonids, dark gray shaded taxa are subterranean species, no shaded taxa are surface water species. Letters next to taxa denote individual tribes: (a) Candonini; (b) Cryptocandonini; (c) Candonopsini; (d) Trapezicandonini; (e) Humphreyscandonini

The larger clade on the tree was composed of two tribes. All except *Cryptocandona smithi* Karanovic & Lee, 2012 belong to the largest Candonidae tribe, Candonini (letter “a”). *Cryptocandona smithi* was its sister taxon. Candonini can be broadly divided into three clades, all with maximum posterior probability. The 10 Lake Baikal candonids alone (light gray shaded group) did not form a monophyletic clade, but clustered with some non‐Baikal species, in particular *Fabaeformiscandona kushiroensis*,* Candona candida*,* C. bimucronata*, and *C. neglecta*. A clade composed of nine species belonging to *Candona*,* Pseudocandona,* and *Typhlocypris* was sister to the previous, mostly Baikal candonids, but this association did not have high posterior probability (0.7). The last group on the tree, consisting of *Earicandona* Karanovic, 2015 and *Fabaeformiscandona* Krstić, 1972, was strongly supported and was sister to the previous two clades.

The results of 18S analysis almost did not differ in topology from the concatenated dataset analysis. On the 18S tree Cryptocandonini had a better support (0.98), and Humphreyscandonini was its sister taxon (with a weak posterior probability). In addition, mostly Baikal candonids and *Candona/Pseudocandona/Typhlocypris* clade had a slightly better support (0.81). Finally, the association between Candonopsini and Trapezicandonini seemed to be the result of the long branch attraction.

The resulting trees of all three 28S fragments analyzes concurred with concatenated and 18S results in terms of general topology, showing a strongly supported division of Candonidae into two clades. However, none of the analyzed fragments resolved the relationships between any of the Baikal candonids or their association with non‐Baikal species, and came out comb‐like with very short branches. Of the three fragments, the vx was the most similar to 18S and concatenated datasets analyzes.

Due to the very limited 16S dataset, the resulting tree did not support partition of Candonidae into two clades, and positioned Trapezicandonini as a sister taxon to Baikal and some other non‐Baikal candonids, but as the Trapezicandonini branch was very long, this union might be a result of the long branch attraction. Similarly to the 28S fragment, the terminal relationships between Baikal candonids were not resolved, and here as well was comb‐like, but with longer branches.

### 3.4 Molecular clock

DUMBE5 analysis indicated no saturation in any of the gene alignments and likelihood ratio test rejected strict molecular clock for the 18S and 16S alignments, while assumed it for all three 28S fragment alignments (Supplement 2). Our test of molecular clock for the concatenated dataset, run under stepping stone algorithm in MrBayes, resulted in differences of 45 (marginal) likelihood units between the clock and no clock runs (no clock mean value = −15945.5, clock mean value = −15987.85), rejecting the strict molecular clock hypothesis, based on the observation that differences exceeding five log likelihoods are usually very strong evidence in favor of a better model (see Kass & Raftery, 1995).

**Figure 5:**
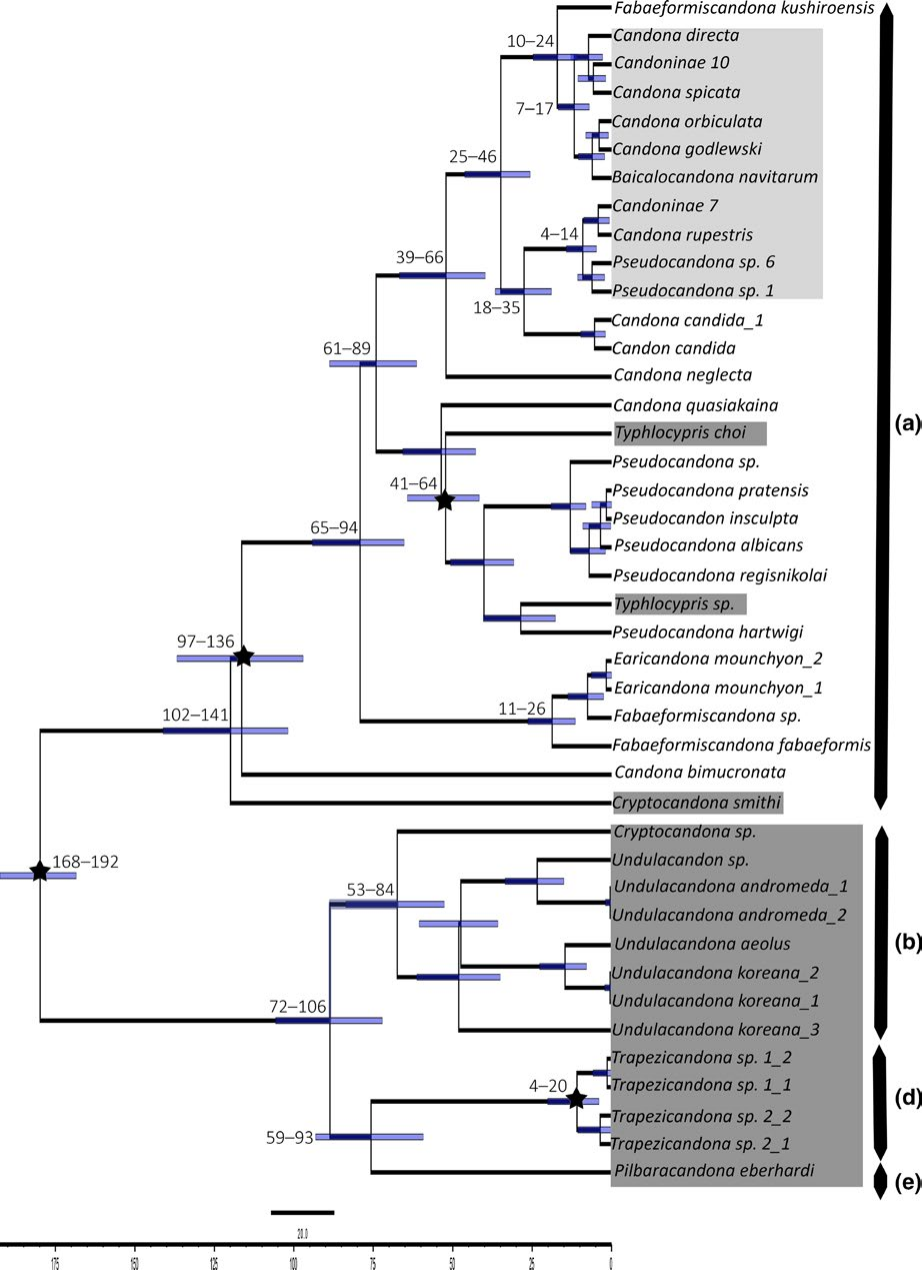
Molecular time divergence estimate tree of the family Candonidae based constructed from 28S rRNA dataset. Stars represent nodes calibrated with fossil record. Numbers above branches represent 95% HPD intervals for particular node heights, while bars represent the same values for all nodes. Light gray shaded taxa are Lake Baikal candonids, dark gray shaded taxa are subterranean species, no shaded taxa are surface water species. Letters next to taxa denote individual tribes: (a) Candonini; (b) Cryptocandonini; (d) Trapezicandonini; (e) Humphreyscandonini

For the concatenated and 18S datasets we used uncorrelated relaxed (lognormal) clock. Calibrated Yule Model was used as a tree prior in strict and the relaxed clock analyzes. All analyzes resulted in similar tree topologies (Figures 4 and 5), with similar posterior probabilities, and they were almost identical to the unconstrained analysis in MrBayes. Differences are minor. For example, the tribe Candonopsini (Figure 4c) resulted as a sister taxon to Cryptocandonini, Humphreyscandonini, and Trapezicandonini. Candonopsini lineage was not recovered on the 28S time tree because of unsuccessful amplification of this region. Divergence time estimates are similar on both trees, with mean values older than the fossil data. For example, the node age for the most recent common recent of *Candona* ssl. was 100+ million years, although the fossil dates 85–75 million years. Similarly, the fossil range of the *Pseudocandona* most recent common ancestor was 28–20 million years, while the estimates of the divergence time (on all datasets) reconstructed this node as 50+ million years. Time trees differed in the 95% confidence intervals for the node heights, in that the intervals for 28S dataset were much narrower than those on the concatenated dataset. Traces analysis in Supplement 3 summarizes some of the BEAST results. Although, estimated sample sizes did not fall below threshold value of 100, in the analysis of the concatenated dataset they were significantly lower than in the 28S analysis.

## 4 DISCUSSION

### 4.1 Divergence time estimates

The fact that both divergence time analyzes produced consistently older estimates than the fossil record suggested is in accordance with previous studies on ostracods based on 18S (Tinn & Oakley, 2008). These authors showed that the molecular divergence rates differ among ostracod lineages, and that molecular time estimates are not always older than the fossil record would suggest. In contrast to our analyzes, they showed that the relaxed clock model aligns fossil and molecular time estimates better than the strict clock. In our study, the strict clock model applied to the 28S dataset analysis resulted in smaller age differences between fossil and molecular divergence time estimates. One of the reasons why the concatenated dataset suggested older dates is that the 18S dataset when analyzed alone (results not included in this study) placed divergence time even further back in the past, so the concatenated dataset reflected a consensus between 18S and 28S rates of evolutions. We agree with Tinn and Oakley (2008) that one of the reasons for the incongruence between molecular and fossil estimates is a problematic taxonomy of fossils, especially in the case of our internal nodes calibrations. As all Candonidae have the same adductor muscle scar imprint, but other shell characters are homoplastic (see above), it is hard to be sure if, for example, the fossil record of *Candona* from the Upper Cretaceous (see Danielopol et al., 2011) represents the ancestral lineage to some of the presently diversified groups or a common ancestor to all *Candona* like ostracods. On the other hand, we believe that *Septacandona* from the Lower Jurassic may indeed represent the oldest known ancestral lineage to all Recent Candonidae. Considering the problems surrounding the taxonomy of fossils, we think that the divergence dates estimates we present here are the best hypothesis at the moment.

The results of our BEAST analysis suggested the existence of at least two Candonidae lineages in Lake Baikal, and potentially two independent colonization events. The concatenated dataset showed that the most recent common ancestor of *Baicalocandona* and a group of Baikal *Candona* species lived about 40 Mya, while that of Baikal *Pseudocandona* and another group of Baikal *Candona* lived 20 Mya. This implies that the former group evolved before Lake Baikal was formed, while the latter may have evolved in some shallow lakes which preceded the formation of today's conditions. However, the latter group is more closely related to a group of typical European *Candona* species than to their Baikal congeners and the most recent common ancestor of this clade appeared 60 Mya. The 28S analysis dated the origin of these two Baikal lineages to a more recent time (12 and 5 Mya respectively), which would allow for the possibility that they both evolved in some shallow lakes in the Lake Baikal region. However, some caution is necessary with this interpretation, as in the period between Upper Miocene and Lower Pliocene (10–5 Mya) a great diversification of Candonidae lineages occurred in the Pannonian basin (Central Europe) after the closure of Paratethys under the conditions of decreased salinity in the Pannonian Sea (see Krstić, 1972). The habitat shift from saline to freshwater may have prompted this diversification, as it apparently happened with the Tethyan amphipods (Hou, Sket, & Li, 2011). This is important because, some of the Pannonian candonids (allocated to various genera described from this fossil record) strongly resemble forms found today in Lake Baikal.

Our results of the 28S divergence time estimates are very similar to those published by Schön and Martens (2012) for the other endemic ostracod group here, *Cytherissa*. Previously, and based on morphological data alone, Danielopol, Olteanu, Löffler, and Carbonel (1990) suggested that the Baikal *Cytherissa* flock originated through several independent radiations; they recognized at least three groups, one with earlier colonization time than the other two. This has been confirmed with the molecular divergence time estimates (Schön & Martens, 2012), which placed the time of this Baikal group diversifications between 8 and 5.3 Mya. Since Schön and Martens (2012) study was based on mitochondrial *COI* gene with general *COI* invertebrate clock (Wilke et al., 2009), and on a much larger sample size of Baikal species, this put more weight on our younger time estimates based on 28S. In general, Baikal ostracods diversification times are highly congruent with other animal groups (Kontula et al., 2003; Stelbrink et al., 2015; Zubakov et al., 1997, etc.).

On the other hand, beside potentially receiving fauna from various parts of the world, Lake Baikal was potentially also the fauna source. For example, the Japanese *Fabaeformiscandona kushiroensis* was deeply nested inside the older Baikal candonid clade on all our unconstrained and on constrained concatenated datasets. However, our 28S analysis placed *F. kushiroensis* ancestral to this Baikal *Candona* lineage, and opened the possibility that one of the Baikal candonid lineages colonized the lake from the East. Other studies based on molecular markers suggest similar scenario to our concatenated datasets for some *Cytherissa* ostracods (see Schön & Martens, 2012) and other groups with species flocks in Lake Baikal: Sculpin fishes have a high diversity in Baikal and one closely related species in Lake Michigan (see Sherbakov, 1999); and an amphipod species found in Finish streams has closest relatives in Baikal (Vainola & Kamaltynov, 1995).

Karanovic and Abe (2010) and Karanovic, Grygier, and Lee (2013) attributed to ancient lakes a role of biodiversity pumps for subterranean habitats in addition to their role as refugia, because their deep and dark benthic environments provide ideal conditions for the evolution of subterranean adaptations. Our resulting trees did not reveal a close connection between subterranean ostracods and those from Lake Baikal, but our sample from the lake was limited. However, our results highlighted a monophyly of tribes which today have almost all representatives in subterranean waters. Their distribution suggests that the most recent common ancestor, which according to the divergence time estimates lived 90‐110 Mya, must have been a widely distributed species, because Trapezicandonini and Cryptocandonini live in Europe and Humphreyscandonini in Australia. This ancestor might have been either a surface freshwater species or a marine one that was widely distributed in Tethys and Parathethys. The oldest Candonidae fossil, *Septacanonda,* was recovered from both marine and brackish sediments (Cabral & Colin, 2002), stipulating that candonids originated in the sea. Colonization of the subterranean waters by this clade might have happened in different periods, but the fact that they all presently live in this environment strongly suggests that the ancestral lineage had good preadaptations for the subterranean mode of life. The tribe Candonini also has some subterranean representatives, but a majority of species live in surface waters. The colonization of subterranean waters under the stress of climate cooling in Pleistocene was suggested for ostracods and other crustacean groups (see review in Danielopol, 1980), and this may be the time of Candonini colonization as well. We believe that by the time Candonini started colonizing subterranean waters, this ecosystem was already inhabited by other candonid lineages, causing strong competition. There is a possibility that the clade consisting of the tribes Trapezicandonini, Cryptocandonini, and Humphreyscandonini invaded subterranean waters from marine environments, which has also already been postulated for unrelated ostracods and other crustacean groups (see Danielopol, 1980). However, supposedly the most recent common ancestor of the genus *Trapezicandona* lived about 6 Mya in cold fresh surface waters (Danielopol, 1968), contradicts this hypothesis.

### 4.2 Phylogenetic position of Baikal candonids

According to Mazepova (1990), the phylogenetic relationship between *Baicalocandona* and the other two Lake Baikal candonid genera, *Candona* and *Pseudocandona*, is unresolved. Results of our analyzes indicate that *Baicalocandona* is closely related to some of the Baikal *Candona* species, but also to *Fabaeformiscandona kushiroensis*, a species recently described from Japan (Hiruta & Hiruta, 2015). On the unconstrained tree, the clade formed by *Baicalocandona* and *Fabaeformiscandona* did not receive a high posterior probability, in contrast to the BEAST results where the posterior probability was very high for the concatenated dataset. The BEAST results of the 28S rRNA analysis suggested the maximum posterior probability for the sister relationship between *Fabaeformiscandona kushiroensis* and *Baicalocandona navitarum,* plus the same group of Baikal *Candona* species. Results may imply that some of the *Candona* species from the Lake Baikal, but also some of the non‐Baikal candonids, should be transferred into *Baicalocandona*, significantly widening the genus distribution. It also may imply that at least some of *Baicalocandona* may need to be transferred to the genus *Candona*. However, the latter is unlikely, because four other candonids, *Candona rupestirs*,* Pseudocandona* sp. 1, sp. *6*, and Candoninae 7, from the lake form a highly supported clade with *Candona candida*,* C. bimucronata*, and *C. neglecta,* all known from Europe or Palearctic in general. *Candona candida* is the type species of the genus, and those four Baikal species form a highly supported subclade, questioning their position within *Candona* as well.

Baikal *Pseudocandona* are defined by a trapezoidal, strongly ornamented shell, and absence of male sexual bristles on the second antenna. The last character was the strongest argument of Bronstein (1947) and Mazepova (1990) to assign all such Baikal species to *Pseudocandona,* since the type species of the genus, *P. insculpta*, lacks those bristles. But since Kaufmann (1900) erected *Pseudocandona*, species with and without male bristles have been assigned to it (see Meisch 2000), recognizing that this is a homoplastic character. This is also clear from our analyzes: *Pseudocandona insculpta* (which lacks sexual bristles) was part of a clade distinct from the Baikal *Pseudocandona* species and it also clustered with a species which possess well‐developed sexual bristles, *P. albicans*, and not with the species lacking the bristles, *P. regisnikolai*.

All resulting trees showed that the number of Candonidae lineages currently recognized in the lake may need to be revised. Unconstrained analyzes and divergence time analysis of the concatenated dataset suggested two lineages: *Baicalocandona* and species currently assigned to *Pseudocandona*, both of which would also include Baikal *Candona* species. On the other hand, a divergence time estimate of the 28S implied four lineages: *Baicalocandona,* two groups of *Candona* species, and species currently assigned to *Pseudocandona*. Based on the current results which rendered high posterior probability for all possible combinations, it is hard to be more decisive on the number of lineages, although morphological diversity of Baikal candonids leans toward a higher number. Nevertheless, morphological and molecular evolutions have been uncoupled in many ancient lake flocks (Martens, 1994). For example, in Baikal amphipods a morphologically extremely diverse family Acanthogammaridae is monophyletic, while morphologically conservative Micruropodidae is paraphyletic (Macdonald et al., 2005).

Schön and Martens (2012) recovered at least four lineages within Baikal *Cytherissa* species flock, but the basal branches remained unresolved, and the authors believe that assignment of all species to one genus underestimates real morphological variability. The above‐mentioned example of homoplasy related to the male sexual bristles is just one of many cases of convergent evolution in candonid ostracods. There are numerous examples from subterranean ostracods from Western Australia (Karanovic, 2007). This is particularly true for the shell shape and ornamentation. Projecting shells of the Baikal candonids onto either of the resulting trees shows little congruence between the shape/ornamentation and phylogeny. Soft parts morphology remains obscure for all Baikal candonids, and further conclusions need to wait detailed taxonomic studies, because the morphology of hemipenis seems to best reflect phylogenetic relationships between candonid lineages (see Karanovic, 2007).

### 4.3 Phylogeny of candonidae

Our analyzes supported five of the eight Candonidae tribes proposed by Karanovic (2007), and molecular phylogeny was almost identical to the morphological one proposed in the same publication. Few differences include the position of the Candonopsini basal to Cryptocandonini, Trapezicandonini, and Humphreyscandonini in the present analyzes vs basal position to Candonini from the morphological data. This may be a result of a long branch attraction (see above), but may also represent true phylogeny. The position of two Cryptocandonini species included in our analyzes within both Candonidae clades was a result of an unresolved taxonomy within *Cryptocandona* Kaufmann, 1900. Karanovic & Lee (2012) and Karanovic & Cho (2017) already pointed out that two *Cryptocandona* species described from South Korea and Japan have isolated position in the genus and should, together with a few other species from Sweden (Ekman, 1908), belong to a yet undescribed genus.

The morphological phylogeny of Candonidae was carried out on the genus level and could not reveal polyphyletic nature of several Candonini genera, although this tribe was in fact the only paraphyletic lineage in Karanovic's (2007) cladistic analysis. The present molecular study showed that the most diverse Candonini genera (*Fabaeformiscandona*,* Candona,* and *Pseudocandona*) are all in fact polyphyletic, which has already been pointed out in various publications (Danielopol et al., 2011; Karanovic, 2005, 2006, 2012 and Namiotko et al., 2014). Specific discussion about the potential reasons and possible solutions of this problem is beyond the scope of the present paper, and taxonomic revision of these genera will be done elsewhere. This would also require a wider taxon sampling.

## CONFLICT OF INTEREST

None declared.

## DATA ACCESSIBILITY

DNA sequences: GenBank accession numbers (XY1–XY100).

## References

[ece33159-bib-0001] Altschul, S. F. , Gish, W. , Miller, W. , Myers, E. W. , & Lipman, D. J. (1990). Basic local alignment search tool. Journal of Molecular Biology, 215, 403–410. 2231712 10.1016/S0022-2836(05)80360-2

[ece33159-bib-0002] Annenkova, N. V. (2013). Phylogenetic relations of the dinoflagellate *Gymnodium baicalense* from Lake Baikal. Central European Journal of Biology, 8, 366–373.

[ece33159-bib-0003] Bronstein, Z. S. (1947). Ostracoda presnyh vod. Fauna SSSR. Rakoobraznye, Tom II, volume 1, Akedemii Nauk SSR, 339 pp.

[ece33159-bib-0004] Cabral, M. C. , & Colin, J. P. (2002). Taxonomie et paléoécologie de nouveaux ostracodes limniques Candonidae dans l'Oxfordien (Jurassique supérieur) du Portugal. Geodiversitas, 24, 61–76.

[ece33159-bib-0005] Castresana, J. (2000). Selection of conserved blocks from multiple alignments for their use in phylogenetic analysis. Molecular Biology and Evolution, 17, 540–552. 10742046 10.1093/oxfordjournals.molbev.a026334

[ece33159-bib-0006] Daneliya, M. E. , Kamaltynov, R. V. , & Väinölä, R. (2011). Phylogeography and systemtics of *Acanthogammarus* s. str., giant amphipod crustaceans from Lake Baikal. Zoologica Scripta, 40, 623–637.

[ece33159-bib-0007] Danielopol, D. L. (1968). Sur l'appartenance probable de certaines fossils au genre Mixta Klie 1938 (Ostracoda, Podocopida). Warszawa: Proc. Int. Paleontol. Union, XXIII Int. Geol. Congr pp. 123–126.

[ece33159-bib-0008] Danielopol, D. L. (1980). An essay to assess the age of the freshwater interstitial ostracods of Europe. Bijdragen tot de Dierkunde, 50, 243–291.

[ece33159-bib-0009] Danielopol, D. L. , Baltanás, A. , Morocutti, U. , & Österreicher, F. (2011). On the need to renew the taxonomic system of the Candoninae (Non‐Marine Ostracoda, Crustacea). Reflexions from an analysis of data using the Yule Process. Geo‐Eco‐Marina, 17, 197–212.

[ece33159-bib-0010] Danielopol, D. L. , Olteanu, R. , Löffler, H. , & Carbonel, P. (1990). Present and past geographical and ecological distribution of Cytherissa (Ostracoda, Cytherideidae) In Danielopol D. L., Carbonel P. & Colin J. P. (Eds.), Cytherissa (Ostracoda) – The Drosophila of Palaeolimnology. Bull.l'Inst. Géol. Bassin d'Acquitaine, 47–48 (pp. 97–116). Bordeaux: Université Bordeaux.

[ece33159-bib-0011] Darriba, D. , Taboada, G. L. , Doallo, R. , & Posada, D. (2012). jModelTest 2: More models, new heuristics and parallel computing. Nature Methods, 9, 772. 10.1038/nmeth.2109 PMC4594756 22847109

[ece33159-bib-0012] Drummond, A. J. , Suchard, M. A. , Xie, D. , & Rambaut, A. (2012). Bayesian phylogenetics with BEAUti and the BEAST 1.7. Molecular Biology and Evolution, 29, 1969–1973. 22367748 10.1093/molbev/mss075 PMC3408070

[ece33159-bib-0013] Ekman, S. (1908). Ostracoden aus den nordschedischen Hochgebirgen. Naturwissenschaftliche Untersuchungen des Sarekgebirges in Schwedisch‐Lappland, 4, 168–198.

[ece33159-bib-0014] Gandolfo, M. A. , Nixon, K. C. , & Crepet, W. L. (2007). Selection of fossils for calibration of molecular dating models. Annals of the Missouri Botanical Garden, 95, 34–42.

[ece33159-bib-0015] Guindon, S. , & Gascuel, O. (2003). A simple, fast and accurate method to estimate large phylogenies by maximum‐likelihood. Systematic Biology, 52, 696–704. 14530136 10.1080/10635150390235520

[ece33159-bib-0016] Heled, J. , & Drummond, A. J. (2011). Calibrated tree priors for relaxed phylogenetics and divergence time estimation. Systematic Biology, 61, 138–149. https://doi.org/10.1093/sysbio/syr087. 21856631 10.1093/sysbio/syr087 PMC3243734

[ece33159-bib-0017] Hidding, B. , Michel, E. , Natyaganova, A. V. , & Sherbakov, D. Y. (2003). Molecular evidence reveals a polyphyletic origin and chromosomal speciation of Lake Baikal's endemic asellid isopods. Molecular Ecology, 12, 1509–1514. 12755879 10.1046/j.1365-294x.2003.01821.x

[ece33159-bib-0018] Hillis, D. M. , & Dixon, M. T. (1991). Ribosomal DNA: Molecular evolution and phylogenetic inference. The Quarterly Review of Biology, 66, 411–453. 1784710 10.1086/417338

[ece33159-bib-0500] Hiruta, S. F. & Hiruta, S. I. (2015). Description of a species of *Fabaeformiscandona* (Ostracoda, Crustacea) from Kushiro Marsh, Hokkaido, Japan, with nearly complete mitochondrial genome sequence. Biodiversity Data Journal, 3, e7074. 10.3897/BDJ.3.e7074 PMC4698455 26751633

[ece33159-bib-0019] Hiruta, S. F. , Kobayashi, N. , Katoh, T. , & Kajihara, H. (2016). Molecular phylogeny of cypridoid freshwater Ostracods (Crustacea: Ostracoda), inferred from 18S and 28S rDNA sequences. Zoological Science, 33, 179–185. 27032683 10.2108/zs150103

[ece33159-bib-0020] Hou, Z. , Sket, B. , & Li, S. (2011). Eocene habitat shift from saline to freshwater prompted Tethyan amphipod diversification. PNAS, 108, 14533–14538. 21844362 10.1073/pnas.1104636108 PMC3167504

[ece33159-bib-0021] Huelsenbeck, J. P. , & Ronquist, F. (2001). MRBAYES: Bayesian inference of phylogeny. Bioinformatics, 17, 754–755. 11524383 10.1093/bioinformatics/17.8.754

[ece33159-bib-0022] Hurvich, C. M. , & Tsai, C. L. (1989). Regression and time series model selection in small samples. Biometrika, 76, 297–307.

[ece33159-bib-0024] Karanovic, I. (2005). On the genus *Typhlocypris* Vejdovský, 1882 (Crustacea: Ostracoda: Candoninae), with description of two new species. Systematics and Biodiversity, 3, 375–406.

[ece33159-bib-0025] Karanovic, I. (2006). Recent Candoninae (Crustacea, Ostracoda) of North America. WA Records and Museum Supplement, 71, 1–75.

[ece33159-bib-0026] Karanovic, I. (2007). Candoninae (Ostracoda) from the Pilbara Region in Western Australia. Crustaceana Monographs, 7, 433.

[ece33159-bib-0027] Karanovic, I. (2012). Recent freshwater ostracods of the world. Berlin‐Heidelberg: Springer. 608 pp.

[ece33159-bib-0028] Karanovic, T. , & Abe, Y. (2010). First record of the harpacticoid genus *Morariopsis* (Crustacea: Copepoda: Canthocamptidae) in Japan, and its zoogeographic implications. Species Diversity, 15, 185–28.

[ece33159-bib-0029] Karanovic, I. , & Cho, J. L. (2017). Phylogenetic position of the East Asian ostracod genus *Undulacandona* within Candonidae with description of four new species from subterranean waters of Korea. Zoological Journal of the Linnean Society, https://doi.org/10.1093/zoolinnean/zlw020.

[ece33159-bib-0030] Karanovic, T. , Grygier, M. J. , & Lee, W. (2013). Endemism of subterranean *Diacyclops* in Korea and Japan, with descriptions of seven new species of the *languidoides*‐group and redescriptions of *D. brevifurcus* Ishida, 2006 and *D. suoensis* Ito, 1954 (Crustacea, Copepoda, Cyclopoida). ZooKeys, 267, 1–76. 10.3897/zookeys.267.3935 PMC3591758 23653520

[ece33159-bib-0031] Karanovic, I. , & Lee, W. (2012). Two new candonid species from South Korea (Ostracoda, Podocopida). Crustaceana, 85, 1633–1656.

[ece33159-bib-0032] Kass, R. E. , & Raftery, A. E. (1995). Bayes factors. Journal of American Statistical Association, 90, 773–795.

[ece33159-bib-0033] Khursevich, G. K. , Karabanov, E. B. , Prokhorenko, A. A. , Williams, D. F. , Kuzmin, M. I. , Fedenya, S. A. , … Kerber, E. A. (2001). Detailed diatom biostratigraphy of the Baikal sediments during the Brunhes chron and climatic factors of species formation. Geologiya i Geofizika, 42, 108–129.

[ece33159-bib-0034] Kontula, T. , Kirilchik, S. V. , & Väinölä, R. (2003). Endemic diversification of the monophyletic cottoid fish species flock in Lake Baikal explored with mtDNA sequencing. Molecular Phylogenetics and Evolution, 27, 143–155. 12679079 10.1016/s1055-7903(02)00376-7

[ece33159-bib-0035] Krstić, N. (1972). Rod Candona (Ostracoda) iz kongerijskih slojeva južnog dela Panonskog Basena [Genus Candona (Ostracoda) from Congeria beds of southern Pannonian Basin − in Serbian with English summary]. Serbian Academy of Sciences and Arts Monograph, 450, Section National Mathematics and Science, Belgrade, 39, 145.

[ece33159-bib-0036] Krstić, N. (2006). Pliocene ostracodes of the Paludinian beds in Pannonian plain. Belgrade: Serbian Part. Her. Nat. Hist. Mus. 409 pp.

[ece33159-bib-0037] Kumar, S. , Stecher, G. , & Tamura, K. (2016). MEGA7: Molecular evolutionary genetics analysis version 7.0 for bigger datasets. Molecular Biology and Evolution, 33, 1870–1874. 27004904 10.1093/molbev/msw054 PMC8210823

[ece33159-bib-0038] Logachev, N. A. (2003). History and geodynamics of the Baikal rift. Geologiya i Geofizika, 44, 391–406.

[ece33159-bib-0039] Macdonald, K. S. III , Yampolsky, L. , & Duffy, J. E. (2005). Molecular and morphological evolution of the amphipod radiation of Lake Baikal. Molecular Phylogenetics and Evolution, 35, 323–343. 15804407 10.1016/j.ympev.2005.01.013

[ece33159-bib-0040] Martens, K. (1994). Ostracod speciation in ancient lakes: A review. Archiv fur Hydrobiologie – Beih Ergeb. limnol., 44, 203–222.

[ece33159-bib-0041] Martens, K. , Coulter, G. , & Goddeeris, B. (1994). Speciation in ancient lakes – 40 years after Brooks. Archiv fur Hydrobiologie – Beih. Ergeb. limnol., 44, 75–96.

[ece33159-bib-0042] Martens, K. , & Savatenalinton, S. (2011). A subjective checklist of the Recent, free‐living, non‐marine Ostracoda (Crustacea). Zootaxa (Monogr.), 2855, 1–79.

[ece33159-bib-0043] Martens, K. , & Schön, I. (1999). Crustacean biodiversity in ancient lakes: A review. Crustaceana, 72, 899–910.

[ece33159-bib-0044] Mats, V. D. (2001). Geological factors of the unique biodiversity of Lake Baikal, in Annotirovannyi spisok fauny ozera Baikal i ego vodosbornogo basseina (Index of animal species inhabiting lake Baikal and its catchment area), vol. 2, book 2: Vodoemy i vodotoki yuga Vostochnoi Sibiri i Severnoi Mongolii (Basins and Channels in South of East Siberia and North Mongolia). Novosibirsk: Nauka pp. 1406—1419.

[ece33159-bib-0045] Mayor, T. Y. , Sheveleva, N. G. , Sukhanova, L. V. , Timoshkin, O. A. , & Kirilchik, S. V. (2010). Molecular phylogenetic analysis of cyclopoids (Copepoda: Cyclopoida) from lake Baikal and its water catchment basin. Russian Journal of Genetics (Translation of Genetika (Moscow, Russian Federation)), 46, 1373–1380. 21261066

[ece33159-bib-0046] Mazepova, G. F. (1990). Rakushkovye rachki (Ostracoda) Baykala (pp. 1–471). Novosibirsk: Akademija Nauk SSSR, Sibirskoe Otdelenie, Limnologicheskii Institut.

[ece33159-bib-0047] Mazepova, G. (1994). On comparative aspects of ostracod diversity in the Baikalian fauna. Archiv fur Hydrobiologie – Beih. Ergeb. limnol., 44, 197–202.

[ece33159-bib-0048] Müller, J. , Oberhänsli, H. , Melles, M. , Schwab, M. , Rachold, V. , & Hubberten, H. V. (2001). Late Pliocene sedimentation in Lake Baikal: Implications for climatic and tectonic change in SE Siberia. Palaeogeography, Palaeoclimatology, Palaeoecology, 174, 305–326.

[ece33159-bib-0049] Namiotko, T. , Danielopol, D. L. , Meisch, C. , Gross, M. , & Mori, N. (2014). Redefinition of the genus *Typhlocypris* Vejdovský, 1882 (Ostracoda, Candonidae). Crustaceana, 87, 952–984. 28090119 10.1163/15685403-00003338 PMC5230036

[ece33159-bib-0050] Palo, J. U. , & Väinölä, R. (2006). The enigma of the landlocked Baikal and Caspian seals addressed through phylogeny of phocine mitochondrial sequences. Biological Journal of the Linnean Society, 88, 61–72.

[ece33159-bib-0051] Palumbi, S. R. , Martin, A. , Romano, S. , McMillan, W. O. , Stice, L. , & Grabowski, G. (1996). The simple fool's guide to PCR. Honolulu: Kewalo Marine Laboratory, University of Hawaii.

[ece33159-bib-0053] Parham, J. F. , & Irmis, R. B. (2008). Caveats on the use of fossil calibrations for molecular dating: A comment on Near et al. The American Naturalist, 171, 132–136. 10.1086/524198 18171158

[ece33159-bib-0054] Popova, S. M. , Mats, V. D. , Chernyaeva, G. P. , Shimaraeva, M. K., Kultchitsky, A. A. , Vorobyova, G. A. , … Shibanova, I. V. (1989). Paleolimnologicheskie rekonstruktsii: Baikal'skaya riftovaya zona (Baikal Rift Zone: Paleolimnological Reconstructions). Nauka: Novosibirsk.

[ece33159-bib-0055] Rambaut, A. , Suchard, M. A. , Xie, D. , & Drummond, A. J. (2014). Tracer v1.6. Retrieved from http://beast.bio.ed.ac.uk/Tracer

[ece33159-bib-0056] Rodríguez, F. , Oliver, J. F. , Marín, A. , & Medina, J. R. (1990). The general stochastic model of nucleotide substitutions. Journal of Theoretical Biology, 142, 485–501. 2338834 10.1016/s0022-5193(05)80104-3

[ece33159-bib-0057] Ronquist, F. , & Huelsenbeck, J. P. (2003). MRBAYES 3: Bayesian phylogenetic inference under mixed models. Bioinformatics, 19, 1572–1574. 12912839 10.1093/bioinformatics/btg180

[ece33159-bib-0058] Ronquist, F. , Teslenko, M. , van der Mark, P. , Ayers, D. L. , Darling, A. , Höhna, S. , … Huelsenback, J. P. (2012). MrBayes 3.2: Efficient Bayesian phylogenetic inference and model choice across a large model space. Systematic Biology, 61, 539–542. 22357727 10.1093/sysbio/sys029 PMC3329765

[ece33159-bib-0059] Schön, I. , & Martens, K. (2012). Molecular analyses of ostracod flocks from Lake Baikal and Lake Tanganyika. Hydrobiology, 682, 91–110.

[ece33159-bib-0060] Schön, I. , Martens, K. , van Doninck, K. , & Butlin, R. K. (2003). Evolution in the slow lane: Molecular rates of evolution in sexual and asexual ostracods (Crustacea: Ostracoda). Biological Journal of the Linnean Society, 79, 93–100.

[ece33159-bib-0061] Schön, I. , Shearn, R. , Martens, K. , Koenders, A. , & Halse, S. (2015). Age and origin of Australian *Bennelongia* (Crustacea, Ostracoda). Hydrobiology, 750, 125–146.

[ece33159-bib-0062] Sherbakov, D. Y. (1999). Molecular phylogenetic studies on the origin of biodiversity in Lake Baikal. Trends in Ecology & Evolution, 14, 92–95. 10322507 10.1016/s0169-5347(98)01543-2

[ece33159-bib-0063] Siveter, D. J. , Briggs, D. E. G. , Siveter, D. J. , & Sutton, M. D. (2010). An exceptionally preserved myodocopid ostracod from the Silurian of Herefordshire, UK. Proceedings of the Royal Society of London B: Biological Sciences, 277, 1539–1544. https://doi.org/10.1098/rspb.2009.2122. 10.1098/rspb.2009.2122 PMC2871837 20106847

[ece33159-bib-0064] Sohn, I. G. (1975). Dunkard Ostracoda – an evaluation In Barlow J. A. (Ed.), The age of Dunkard (pp. 265–280). West Virginia Geological and Economic Survey, Morgantown: Proc. First I. C. White Mem. Symp.

[ece33159-bib-0065] Sohn, I. G. (1977). Muscle scars of Late Paleozoic freshwater ostracodes from West Virginia. Journal of Research, US Geological Survey, 5, 135–141.

[ece33159-bib-0066] Starobogatov, Y. I. , & Sitnikova, T. Y. (1992). Process of the speciation in gigantic lakes – ecological investigation of Baikal and its region. Irkutsk, 1, 18–35 (in Russian).

[ece33159-bib-0067] Stelbrink, B. , Shirokaya, A. A. , Clewing, C. , Sitnikova, T. Y. , Prozorova, L. A. , & Albrecht, C. (2015). Conquest of the deep, old and cold: An exceptional limpet radiation in Lake Baikal. Biology Letters, 11, 1–4. 10.1098/rsbl.2015.0321 PMC4528446 26202427

[ece33159-bib-0068] Takheev, V. V. (2000). Ocherki o bokoplavakh ozera Baikal (sistematika, sravnitelnaya ekologiya, evolutsiya). Irkutsk: Izdadelstvo Irkutskogo Universiteta.

[ece33159-bib-0069] Thompson, J. D. , Higgins, D. G. , & Gibson, T. J. (1994). Clustal‐W – improving the sensitivity of progressive multiple sequence alignment through sequence weighting, position‐specific gap penalties and weight matrix choice. Nucleic Acid Research, 22, 4673–4680. 10.1093/nar/22.22.4673 PMC308517 7984417

[ece33159-bib-0070] Timoshkin, O. A. (2001). Lake Baikal: Diversity of fauna, problems of its immiscibility and origin, ecology and “exotic” communities In Timoshkin O. A. (Ed.), Index of animal species inhabiting Lake Baikal and its catchment area (pp. 74–113). Novosibirsk: Nauka.

[ece33159-bib-0071] Tinn, O. , & Oakley, T. H. (2008). Erratic rates of molecular evolution and incongruence of fossil and molecular divergence time estimates in Ostracoda (Crustacea). Molecular Phylogenetics and Evolution, 48, 57–167. 10.1016/j.ympev.2008.03.001 18482852

[ece33159-bib-0072] Triebel, E. (1963). Ostracoden aus dem Sannois und jüngeren Schichten des Mainzer Beckens: 1. Cyprididae Senckenberg lethaea, 44, 157–207.

[ece33159-bib-0073] Vainola, R. , & Kamaltynov, R. (1995). Allozyme studies on the evolutionary diversity of Baikalian amphipode crustaceans, and their relationship too European escapee species *Pallasea quadrispinosa* The second Vereshchagin Baikal conference (Abstr.) (pp. 40–41). Irkutsk, Russia.

[ece33159-bib-0074] Warnock, C. M. , Parham, J. F. , Joyce, W. G. , Lyson, T. R. , & Donoghue, P. C. J. (2014). Calibration uncertainty in molecular dating analyses: There is no substitute for the prior evaluation of time priors. Proceedings of the Royal Society B, 282, 1–10. 10.1098/rspb.2014.1013 PMC4262156 25429012

[ece33159-bib-0075] Wiens, J. J. (2003). Missing data, incomplete taxa, and phylogenetic accuracy. Systematic Biology, 52, 528–538. 12857643 10.1080/10635150390218330

[ece33159-bib-0076] Wiens, J. J. , & Moen, D. S. (2008). Missing data and the accuracy of Bayesian phylogenetics. Journal of Systematics and Evolution, 46, 307–314.

[ece33159-bib-0077] Wilke, T. , Schultheiß, R. , & Albrecht, C. (2009). As time goes by: A simple fool's guide to molecular clock approaches in invertebrates. American Malacological Bulletin, 27, 25–45.

[ece33159-bib-0078] Xia, X. (2013). DAMBE5: A comprehensive software package for data analysis in molecular biology and evolution. Molecular Biology and Evolution, 30, 1720–1728. 23564938 10.1093/molbev/mst064 PMC3684854

[ece33159-bib-0080] Yamaguchi, S. (2003). Morphological evolution of cytherocopine ostracods inferred from 18S ribosomal DNA sequences. Journal of Crustacean Biology, 23, 131–153.

[ece33159-bib-0081] Zubakov, D. Y. , Sherbakov, D. Y. , & Sitnikova, T. Y. (1997). Phylogeny of the endemic mollusks inferred from partial nucleotide sequences of the CO1 mitochondrial gene. Molecular Biology, 31, 935–939.

