# Supplementary material for: Phylogenetic position and age of Lake Baikal candonids (Crustacea, Ostracoda) inferred from multigene sequence analyzes and molecular dating
Source: Ecol Evol. 2017 Aug 1;7(17):7091–103. doi: 10.1002/ece3.3159 (PMC5587501; doi:10.1002/ece3.3159)
Supplement: Supplementary file 2 [file ECE3-7-7091-s002.docx]

| **Marker** | **N of terminals** | **L of alignment** | **Conserved sites** | **Parsimony informative sites** | **Model**** | **-lnL** | **base f^@^** | **rate f^^^** | **p-inv** | **gamma shape** |
| --- | --- | --- | --- | --- | --- | --- | --- | --- | --- | --- |
| *18S rRNA* | 50 | 1042 | 806 | 176 | GTR+I+G | 4061.4315 | 0.276 0.203 0.273 0.248 | 2.03 5.48 3.59 1.12 7.45 1.00 | 0.499 | 0.705 |
| *28S rRNA (df)* | 34 | 455* | 365 | 71 | TIM2+I+G | 1511.2308 | 0.268 0.201 0.293 0.239 | 2.81 4.02 2.81 1.00 8.24 1.00 | 0.613 | 0.895 |
| *28S rRNA (ef)* | 40 | 660* | 378 | 164 | TVM+G | 3319.8486 | 0.241 0.227 0.301 0.231 | 1.63 4.24 3.41 0.52 4.24 1.00 | N/A | 0.542 |
| *28S rRNA (vx)* | 41 | 606* | 482 | 98 | TIM2ef+I+G | 2059.0618 | equal | 1.65 7.33 1.65 1.00 10.6 1.00 | 0.532 | 0.612 |
| *16S rRNA* | 21 | 554 | 316 | 133 | TPM2uf+I+G | 3013.5114 | 0.369 0.191 0.097 0.344 | 1.97 10.69 1.97 1.00 10.69 1.00 | 0.277 | 0.392 |
| *concatenated data set* | 50 | 3302 | 2191 | 686 | GTR+I+G | 15374.35 | 0.28  0.217 0.254 0.253 | 1.88 4.11 3.52 0.79 7.32 1.00 | 0.397 | 0.657 |
